# Notes on Diseases of the Skin

**Published:** 1886-10

**Authors:** A. R. Davidson

**Affiliations:** Prof. Medical Chemistry and Dermatology, Niagara University


### *NOTES ON DISEASES OF THE SKIN.*

By A. R. DAVIDSON, M. D.,

Prof. Medical Chemistry and Dermatology, Niagara University.

Cocaine in Herpes Zoster.—Weissenberg, in the *Allg. Med. Cent. Zeitung*, recommends the application of a five per cent. solution of cocaine, every two hours, over the seat of the eruption. He reports one case in which extremely satisfactory results followed. The burning pain was immediately relieved, and at the end of twelve days no trace of the eruption remained. The cocaine, in addition to its anæsthetic properties, was thought to have hastened cicatrization. I have not had an opportunity of trying this. By far the best local treatment, in my experience, is the application of fluid extract of belladonna all over the eruptions. Several applications should be made in succession, each coat being allowed to dry before another is made. Over these a thick coat of collodion should be applied. As Piffard says, apparent abortion of the eruption will sometimes take place under these applications.

HYPERIDROSIS.—The German army surgeons, instead of the salicylic powder, formerly employed, now recommend salicylic suet, as a remedy for sore feet, etc., due to excessive sweating. It is composed of one to two parts of pure salicylic acid to ten parts of best mutton suet.

---

ICTHYOL IN SKIN DISEASE.—This substance has been highly praised as a remedy of importance, and has been used, to some extent, in Germany and England. The writer in 1883 found Sangster at the Charing Cross Hospital, London, using it frequently—with moderately successful results. Stelwagon, of Philadelphia, has been experimenting with it, and, in a paper read at the recent meeting of the American Dermatological Association, made the following comments upon it. In a small proportion of cases of rosacea and acne vulgaris a ten to twenty per cent. preparation was found beneficial; in eczema it was valueless and irritating; in furunculus it acted with good results in three cases, when applied as a twenty per cent. plaster; in the fourth case it had no effect. It was of service in psoriasis, and also in a case of lupus erythematosus; in favus it was used without effect.

Unna, whose opinion always carries great weight, considers the action of icthyol as similar to that of pyrogallol, chrysarobin, etc.; that is, they are reducing agents, drawing oxygen from the tissues. He considers it of great use in *rosacea*, of which disease he recognizes two forms, one approaching to a usual erythema and eczema, with bright red color, smooth or easily scaly skin, without comedones or acne; the other consisting of a papular acne upon blueish, red swollen base. In the first form a low strength of icthyol is used; in the second the drug is used liberally, both externally and internally. Under its use, in either form, a rapid paling of the surface takes place, a thinning of the epidermis and disappearance of the lesions. In *acne*, whether pustular, papular or indurated, it is very useful and should be employed in full doses, both outwardly and inwardly. Lichen urticatus is very promptly cured by the internal use of the drug. *Urticaria*,

*erythema multiforme et nodosum*, *herpes*, *labialis et progenetalis*, *zoster* and the *parasitic diseases*, he reports, as very favorably influenced by the application of from ten to fifty per cent. ointment, and that most cases of chronic, obstinate relapsing skin diseases are benefited by the internal administration of the drug. He employs icthyol ammonia sulphate, adult dose, five to ten drops, three times a day.

Icthyol is obtained by the dry distillation of a bituminous shale, found in the Tyrol, which contains paleozoic fossil fish. It is from this fact that the so-called oil takes its name (Greek, *icthus*, a fish). Analysis shows it to contain carbon, hydrogen, nitrogen, oxygen, and from two to five per cent. sulphur, and a trace of phosphorous. Its reaction is slightly alkaline. It is completely soluble in a mixture of ether and alcohol and forms an emulsion with water.

---

TINEA TONSURANS.—Van Harlingen, (*Medical Times*, Aug. 20th), gives the following new formulæ out of the multitude which are constantly appearing in the journals :

| | | | |
|---|---|---|---|
| ℞ | Potassii iodidi, - - - | ʒss. | |
| | Liq. Potassæ, - - - - | ℥i. | M. |

The hair is to be closely clipped and this sopped onto the scalp, with a pledget of lint, once daily ; when dry the following solution should be applied at the same points :

| | | | |
|---|---|---|---|
| ℞ | Hydrargyr ; bichlor, - - | gr. iii. | |
| | Aquæ, - - - - - | ℥i. | M. |

The theory is that the liquor potassa dissolves the fatty matter on the surface, and permits the penetration of the iodide of potassium into the follicles. Here it is reached by the corrosive chloride, and iodide of mercury is formed just where it is wanted as a parasiticide. We fear, however, that this ingenious compound application will fail in practice.

A somewhat similar preparation is the following :

| | | | |
|---|---|---|---|
| ℞ | Hydrargyr. chlor., mit., - - | ʒi. | |
| | Tinct. iodinii, - - - - | ℥i. | M. |

Let it stand a few days, shaking frequently; paint on with a brush. "One or two applications, it is said, will suffice."

The following is an old friend in a new combination:

| | | | |
|---|---|---|---|
| ℞ | Thymol, - - - - | ℥i. | |
| | Chloroform, - - - - | ℥ss. | |
| | Ol. olivæ ad., - - - - | ℥ii. | M. |

Apply with a camel's hair brush, after cleansing the part well.

Very satisfactory results can usually be attained by the use of turpentine and tincture of iodine.

The hair being closely cut over the affected part, oil of turpentine is rubbed into the patch, with the fingers, for several minutes. This is to be followed by an application with a camel's hair brush, of tincture of iodine.

The following is said to be a cleanly and efficient remedy in ring-worm of the thigh (eczema marginatum):

| | | | |
|---|---|---|---|
| ℞ | Acid salicylici, - - - - | gr. L. | |
| | Icthyolici, - - - - | ʒss. | |
| | Spirit vini. rect., - - - | ℥i. | M. |

Rub in with a stiff brush twice daily, and afterwards dust with starch.

That the Tricophyton will not always succumb to the treatment suggested is sufficiently well known to most of us, and, apropos of this, Dr. Brocq, of Paris, in a communication to the *Journal of Cutaneous* and *Venereal Diseases*, says: "A fact which has struck me since I have been keeping up with the work done by Americans and English, is the facility with which you radically cure parasitic affections of the scalp, favus and tinea tonsurans, by the use of parasiticides alone, without having recourse to epilation, while in France we obtain, by your method only apparent and transitory cures. If we do not employ epilation we always have relapses."

The American tineæ are not more susceptible than the French, but we do not see as many severe cases. In mild cases, in which the fungus has not penetrated deeply into the hair follicles, almost any antiparasitic application will succeed, but if the tricophyton has effected a lodgment in the hair follicle and passed into the hair shaft, a cure cannot be effected without epilation, all loose hairs and all broken ones should be removed, not only do we thus remove an immense amount of fungus with the extracted hair shaft, but are the better enabled to apply the parasiticide to the seat of the fungus, and the application should follow each epilation.

---

THE LEPROSY BACILLUS. — Dr. Lindsay Stevens, (*British Medical Journal*), has carefully examined a portion of affected skin, excised during life, Gram's method being employed. Bacilli were present in enormous numbers, and were situated in roundish masses of granulation tissue, as well as in more diffuse infiltration of round cells, but scarcely, if at all, in the more normal portions of the sections. None were met with in the epidermis. The bacilli were contained within swollen lymphoid cells, and were also free, these general arrangements being often suggestive of their being contained within the lymphatic spaces. No bacilli were found in the blood vessels. When examined by very high powers, (1,600 diameters) they were seen to be fine rods, often sharply pointed at each extremity, and almost all of them contained small rounded spores, from three to five in number, giving a beaded appearance.

---

A MODERN SAINT AND MARTYR.—Upon the island of Molonai are confined all the lepers from the neighboring Sandwich group. For many years, shut away from all civilized and healthy humanity, Father Damen has been a willing prisoner, ministering to the spiritual and physical wants of these poor unfortunates. For a long time he remained in good health, but at last he has succumbed, and is now to be numbered among the living dead, buried forever in that awful sepulchre, the Leper's Island. In a letter, written recently, he says: "Impossible for me to go any longer to Honolulu, on account of the leprosy breaking out on me. The microbes have finally settled themselves in my left leg and my ear, and one eyebrow begins to fall. I expect to have my face soon disfigured. Having no doubt, myself, of the true character of the disease, I feel calm, resigned and happier among my people. Almighty God knows what is best for my sanctification, and with that conviction I say daily, *Fiat voluntas tua.*" Where is the heroism which will vie with this ?

---

TREATMENT OF PSORIASIS—Dr. Guibout (*Gazette des Hospitaux*) prefers oil of cade, well rubbed in, and alkaline baths every day or so. This treatment to be continued until the pigmentation, due to the disease, has disappeared.

If, for any reason, he cannot use the oil cade, he employs a 5 to 15 per cent. pyrogallic acid ointment, having it applied twice a day, and directing the patient to take a daily bath.

The eruption of psoriasis may be removed in many cases by the above treatment, which is not new. Oil cade and oil turpentine, equal parts of each, acts more quickly. Medicated collodion applications are altogether preferable to ointments. The following formula is commended :

| | |
|---|---|
| Chrysophanic acid, - - | gr. xxx. |
| Salicylic acid, - - - | gr. xx. |
| Collodion, - - - - | ℥i. |

Pyrogallic acid (one-half the strength) may be used in the above formula instead of the chrysophanic, but, in my experience, the action of the latter is more prompt and pronounced.

External treatment should always be supplemented with internal medication.

